# Reduced Pro-Inflammatory Cytokines after Eight Weeks of Low-Dose Naltrexone for Fibromyalgia

**DOI:** 10.3390/biomedicines5020016

**Published:** 2017-04-18

**Authors:** Luke Parkitny, Jarred Younger

**Affiliations:** Department of Psychology, University of Alabama at Birmingham, Birmingham, AL 35205, USA; lukeparkitny@interleuk.in

**Keywords:** fibromyalgia, inflammation, immune, cytokine, naltrexone, LDN, low-dose

## Abstract

Fibromyalgia (FM) is a complex, multi-symptom condition that predominantly affects women. The majority of those affected are unlikely to gain significant symptomatic control from the few treatments that are approved for FM. In this 10-week, single-blind, crossover trial we tested the immune effects of eight weeks of oral administration of low-dose naltrexone (LDN). We enrolled eight women with an average age of 46 years, symptom severity of 62 out of 100, and symptom duration of 14 years. We found that LDN was associated with reduced plasma concentrations of interleukin (IL)-1β, IL-1Ra, IL-2, IL-4, IL-5, IL-6, IL-10, IL-12p40, IL-12p70, IL-15, IL-17A, IL-27, interferon (IFN)-α, transforming growth factor (TGF)-α, TGF-β, tumor necrosis factor (TNF)-α, and granulocyte-colony stimulating factor (G-CSF). We also found a 15% reduction of FM-associated pain and an 18% reduction in overall symptoms. The findings of this pilot trial suggest that LDN treatment in fibromyalgia is associated with a reduction of several key pro-inflammatory cytokines and symptoms. The potential role of LDN as an atypical anti-inflammatory medication should be explored further.

## 1. Introduction

Fibromyalgia (FM) is a multi-symptom condition with a worldwide prevalence of around 3% [1]. Affected individuals are predominantly female and experience widespread musculoskeletal pain and a range of concomitant symptoms, such as cognitive problems, fatigue, impaired sleep, gastrointestinal discomfort, and mood dysregulation [2]. The pathophysiology of FM may involve sensitization of the central nervous system [3,4], which manifests as a pro-algesic shift in responses to stimuli.

Various medications have been used to treat FM, but only the serotonin-norepinephrine reuptake inhibitors duloxetine and milnacipran, and the gabapentinoid pregabalin, are approved for this purpose in the United States. Outside of the US, these medications are generally used off-label as treatments for fibromyalgia. However, recent reviews suggest that these medications are not likely to offer a substantial improvement in symptoms for the majority of individuals with FM [5,6,7,8].

To help treat individuals who do not respond to the approved fibromyalgia medications, off-label prescriptions are common in the United States. One medication that has been used off-label for the treatment of FM is naltrexone hydrochloride. When used in low doses, this medication is often called low-dose naltrexone (LDN) [9]. Classically, naltrexone is exploited for its opioid antagonist properties, but two small clinical trials conducted by our group suggest that naltrexone may be beneficial in reducing the symptoms of FM [9,10,11]. In both trials, a 4.5 mg dose of LDN was administered orally every night. In both studies, we found that the reduction in FM pain was significantly greater in the LDN than in the placebo condition [10,11]. In addition, we found that an individual’s baseline erythrocyte sedimentation rate (ESR), a marker of inflammation, was strongly correlated with their response to LDN [9,10]. Combined with evidence that LDN administration in individuals with Crohn’s disease is associated with reductions in inflammatory markers [12], our results suggest that LDN may possess immunomodulatory properties.

The goal of this study was to test the hypothesis that LDN reduces inflammation in FM. The primary outcome in this 10-week study was a reduction in plasma biomarkers of inflammation between the two-week baseline period (BL) and the period encompassing the final two weeks of the LDN administration phase (Drug). In addition, we tested for a reduction in pain severity and a reduction in overall symptoms severity between BL and Drug phases.

## 2. Experimental Section

### 2.1. Study Design

This pilot study was a 10-week, single-blind, crossover investigation of the immune effects of LDN in women with FM. Each participant first took part in a two-week baseline phase that was immediately followed by an eight-week LDN administration phase. While participants were told they could receive the placebo at any time during the protocol, no placebo capsules were actually administered. In both phases, daily symptoms were collected twice daily and blood samples were collected twice weekly.

### 2.2. Participant Recruitment and Consent

We recruited consenting women aged 18 to 65 from communities near Palo Alto, California, USA. To be eligible for the study, individuals were required to meet the American College of Rheumatology 2010 diagnostic criteria for primary fibromyalgia [2]. These criteria require the presence of widespread pain, a minimum symptom severity and duration, and the exclusion of any differential diagnoses. Individuals were not eligible if they were pregnant or breastfeeding, using opioid analgesics, regularly using anti-inflammatory medications, had a known adverse reaction to naltrexone, unable to refrain from alcohol during the study, diagnosed with a rheumatological or autoimmune conditions, acutely ill, or diagnosed with a significant psychological comorbidity that would interfere with study participation. Individuals were also ineligible if, at baseline, they had any of the following: baseline body temperature higher than 100 °F (37.8 °C), erythrocyte sedimentation rate (ESR) over 60 mm/h, c-reactive protein (CRP) greater than 3.0 mg/dL, positive rheumatoid factor (RF), antinuclear antibody (ANA) greater than 1:80, or a Hospital Anxiety and Depression Scale (HADS) greater than 16. The trial, including informed consent of all individuals, was conducted in accordance with a protocol approved by the Stanford University Institutional Review Board and was registered with the National Institutes of Health ClinicalTrials.gov registry (NCT02107014).

### 2.3. Medication Administration Protocol

During the medication administration phase, each participant self-administered LDN at a 4.5 mg oral dose, taken in capsule form, at least one hour before going to bed at night. A change to a lower dose of 3.0 mg was made available to the participant if they experienced unpleasant adverse effects at the standard dose. Study medication was prepared by Preuss Pharmacy (Menlo Park, CA, USA) using gelatin capsules, a microcrystalline filler (Avicel, FMC BioPolymer, Rockland, ME, USA), and a non-caloric sweetener. LDN capsules included 4.5 mg Naltrexone HCl per capsule. Participants were provided a two-week supply at a time. Compliance was monitored by asking individuals to return used bottles with any unused medication and by a daily log.

### 2.4. Outcome Data Collection

Expressed plasma markers of inflammation were measured in blood samples that were collected twice weekly over the 10-week study period. Study visits were scheduled within a two-hour window to minimize potential bias associated with diurnal cycles of cytokine expression [13]. Participants attended the Stanford Clinical and Translational Research Unit, where blood was drawn using a 21 or 23 gauge needle from a vein in the anterior cubital fossa or dorsal surface of the hand into an ethylenediaminetetraacetic acid (EDTA)-K2-coated vacutainer tube. The site of venipuncture was alternated between the left and right side to minimize participant discomfort. Plasma was promptly separated from whole blood by centrifugation and stored at −80 °C until analysis. Vital signs were collected at each visit to monitor for signs of emergent acute illness.

Plasma markers of inflammation were quantified by the Human Immune Monitoring Center at Stanford University using a modified standard protocol. Human 63-plex immunoassays (eBiosciences, San Diego, CA, USA) were used according to the manufacturer’s recommendations with modifications as follows: Briefly, beads were added to a 96 well plate and washed. Samples were added to the plate containing mixed antibody-linked beads and incubated at room temperature for 1 h, followed by overnight incubation at 4 °C. Following the overnight incubation plates were washed and then biotinylated detection antibodies were added for a period of 75 min at room temperature. Plates were washed and streptavidin-PE was added. After incubation for 30 min at room temperature, a wash was performed and a reading buffer was added to the wells. Each sample was measured in duplicate. Plates were read using a Luminex 200 instrument (Luminex Corporation, Austin, TX, USA) with a lower bound of 50 beads per sample per cytokine. Custom assay control beads by Radix Biosolutions (Georgetown, TX, USA) were added to all wells. All plate washes were done using a Biotek EL ×405 select deep well washer (BioTek, Winooski, VT, USA). Cold and room temperature incubation steps were performed on an orbital shaker at 500–600 rpm [14].

Symptom data were collected using SurveyToGo software (Dooblo, Kefar Sava, Israel) on a hand-held 7-inch Android tablet. Participants completed the survey prior to retiring to bed at night. Daily pain and overall symptoms were scored on 0–100 visual analog scales (VAS) in response to “how would you rate your general level of pain today?” and “overall, how severe have your fibromyalgia symptoms been today?” respectively. LDN tolerability was measured as the VAS response to “how well are you tolerating the medication?” A daily log of medication dose compliance was taken as the responses to the questions “have you taken your capsule for tonight?” and “did you take your capsule last night?” Compliance with medication was also monitored by asking participants to return the dispensed medication vials. Additional questions were used to collect data on other specific symptoms (e.g., headaches, joint pain, and stress), but those data were not analyzed in this study.

### 2.5. Data Analyses

All statistical analyses were conducted using IBM SPSS Statistics software v21 (Amonk, NY, USA) [15]. Due to the longitudinal, repeated-measures nature of the data, study outcomes were tested using linear mixed models (LMM). Univariate models were used to test for differences in cytokine concentrations, pain, and overall symptoms between BL and Drug phases. The participant identification code was used as the subject nesting variable, the study day as the repeat measures index, and study period (BL or Drug) as the fixed independent factor. Model parameters were estimated using the restricted maximum likelihood (REML) approach and the covariance type was selected based on Akaike’s information criterion (AIC) and Schwarz’s Bayesian criterion (BIC). A compound symmetry covariance type was found to best fit the data and was used for all analyses of repeat-measures data. Parameter estimation was conducted using the default setting. For all analyses we set the family-wise error at 5%, yielding a corrected *p*-value of 0.017, according to the method proposed by Benjamini and Hochberg [16].

## 3. Results

### 3.1. Participants

Data were collected between February and July 2014. Nine women fulfilled the inclusion criteria, but one withdrew from the study prior to commencing LDN because she was relocating interstate and, thus, was unable to comply with all study requirements. All presented analyses were conducted on data obtained from the eight women who completed the study. The participant demographic and relevant clinical information are provided in Table 1.

Three LDN doses were missed over the course of the study, yielding a >99.5% compliance rate. No individuals dropped out of the study due to side effects of LDN. The overall self-reported tolerability of LDN was 84.8% ± 24.4%. During the study, one individual reported an acute exacerbation of pre-existing anxiety and one individual reported an exacerbation of pre-existing, non-specific gastrointestinal issues. Both of these side-effects were judged as unlikely to be associated with the administration of LDN.

### 3.2. Main Results

**Figure 1:**
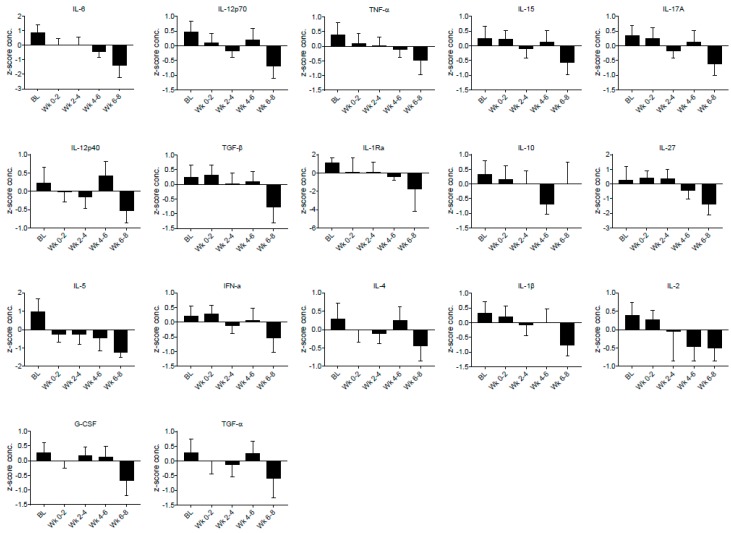
The average plasma concentrations of each cytokine found to be significantly reduced between baseline (BL) and Drug. On the *y*-axis, data are presented as the mean and 95% confidence intervals of within-person *z*-score standardized concentrations, as this provides the most accurate representation of change across the study. The *x*-axis shows the time in blocks of two weeks, starting with baseline (BL).

**Figure 2:**
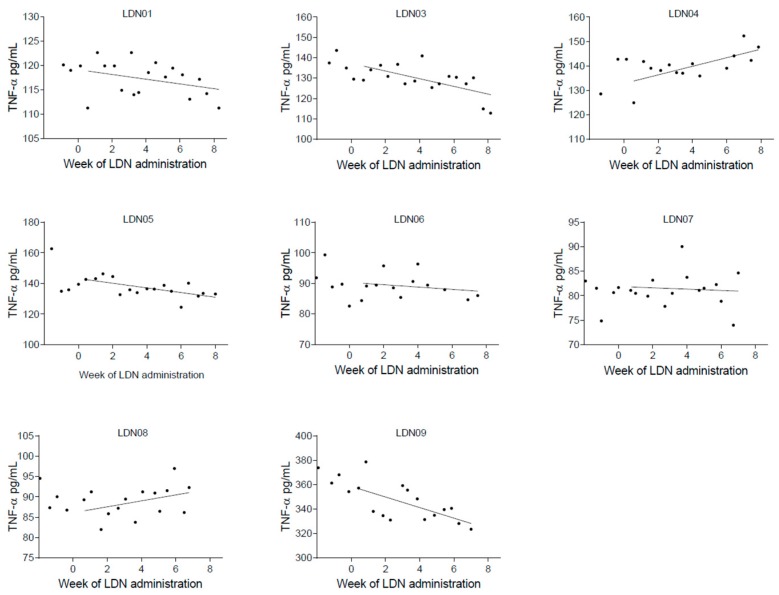
The plasma concentrations of TNF-α measured in samples obtained from each participant during the study. On the *y*-axis, the concentration is shown in pg/mL. On the *x*-axis, time is shown in weeks with zero signifying the commencement of LDN administration. A linear regression line is fitted to the 0–8 week time period.

In our primary analyses, we found the following inflammatory plasma markers to be significantly reduced at Drug compared to BL: IL-1β, IL-1Ra, IL-2, IL-4, IL-5, IL-6, IL-10, IL-12p40, IL-12p70, IL-15, IL-17A, IL-27, IFN-α, TGF-α, TGF-β, TNF-α, and G-CSF (Table 2). The concentrations of these cytokines are presented, for each two-week block, in Figure 1. In addition, as a demonstration of individual cytokine levels over the study period, we present each individual’s TNF-α concentrations for the entire study period in Figure 2. We also found a significant reduction of FM-associated pain (15%), and overall symptoms (18%) at Drug compared to BL.

## 4. Discussion

The goal of this study was to test if LDN administration is associated with reduced markers of inflammation in FM. We found that, after eight weeks of LDN administration, plasma levels of a range of broadly pro-inflammatory cytokines were decreased. In addition, we found that participants reported less pain and symptoms following LDN. Combined, these results support the hypothesis that LDN may help chronic pain conditions, such as fibromyalgia, by acting as an atypical anti-inflammatory medication.

FM is not a classical inflammatory or immune-mediated condition, but the immune system is thought to be a part of its complex pathophysiology [9,17]. However, existing literature on inflammatory abnormalities in FM was insufficient to allow us to predict which specific cytokines would respond to LDN [18]. Therefore, we examined a large array of cytokines. After correcting for multiple comparisons, we found that the cytokines most suppressed by LDN are known to promote nociception, allodynia, and hyperalgesia, including TNF-α, IL-1β, IL-2, IL-6, IL-15, and IL-17 [19,20].

### Study Strengths and Limitations

The three main limitations of this study were the small sample size, the absence of a control group, and the short duration. First, while our longitudinal design gave us sufficient statistical power to detect effects in our cohort, it is not clear how generalizable these findings are to the wider FM population. In particular, we do not how well the findings of our LDN studies translate to men. Second, because we did not use a control group, we cannot conclusively conclude that LDN was solely responsible for the magnitude of the immune change observed in this study. Finally, the short length of the study also meant that we may not have observed the maximal effects of LDN, as previous reports suggest that clinically-beneficial responses develop over months [9]. All of these limitations can be addressed by the conduct of larger, placebo-controlled trials.

We also recognize that some of the cytokines that significantly changed over the course of the study were not expressed by all individuals. However, IL12p40, IL-12p70, TNF-α, IL-4, IL-15, IL-17A, G-CSF, and IFN-α were expressed by all study participants and decreased over the drug administration period.

## 5. Conclusions

The findings of this pilot trial provide early evidence that LDN treatment in FM may be associated with a reduction of several key pro-inflammatory cytokines. The potential role of LDN as an atypical anti-inflammatory medication should be explored further.

## Figures and Tables

**Table 1:** Individual participant demographic and clinical data for baseline (screening) and key study periods. Including erythrocyte sedimentation rate (ESR), c-reactive protein (CRP), hospital anxiety and depression scale (HADS), and fibromyalgia impact questionnaire (FIQ).

Variable	LDN01	LDN03	LDN04	LDN05	LDN06	LDN07	LDN08	LDN09
**Age, years**	35	57	31	47	40	56	36	63
**Ethnicity**	Caucasian	Caucasian	Caucasian	Caucasian	African American	Hispanic/Latina	Hispanic/Latina	Caucasian
**Employment status**	Full-time	Disability	Part-time	Part-time	Part-time	Student/Not employed	Part-time	Part-time
**Baseline ESR, mm/h**	3	14	11	25	2	25	2	55
**Baseline CRP, mg/dL**	<0.2	<0.2	<0.2	<0.2	<0.2	<0.2	<0.2	1.5
**Baseline HADS depression**	1	7	1	11	7	1	11	10
**Baseline HADS anxiety**	8	6	3	8	14	2	10	10
**Baseline FIQ score**	29	76	63	64	79	59	58	72
**Duration of FM symptoms, years**	8	35	16	8	4	12	10	20
**BL pain, mean ± s.d.**	27 ± 11	49 ± 16	52 ± 12	69 ± 7	52 ± 15	73 ± 7	40 ± 20	41 ± 22
**Drug, mean ± s.d.**	28 ± 14	23 ± 7	40 ± 9	76 ± 5	43 ± 13	44 ± 10	56 ± 11	41 ± 24
**BL overall symptoms, mean ± s.d.**	25 ± 16	57 ± 7	64 ± 10	67 ± 6	52 ± 15	73 ± 7	53 ± 17	48 ± 15
**Drug overall symptoms, mean ± s.d.**	31 ± 12	27 ± 7	42 ± 9	72 ± 2	45 ± 11	40 ± 16	67 ± 8	43 ± 26

**Table 2:** Study primary outcomes (cytokines) and secondary outcomes (pain and overall symptoms). All tested cytokines are arranged in order of increasing *p*-values; those found to be significant at the corrected α = 0.017 are highlighted in gray. As a test of generalizability, column two indicates the number of individuals who expressed higher than the lowest detectable concentration (column three) in at least 50% of their collected samples. Columns four and five show the mean (and standard error) of the cytokine concentrations at the two key study time points. The last two columns show the results of the linear mixed models (LMM) analyses. IL = interleukin, TNF = tumor necrosis factor, TGF = transforming growth factor, IFN = interferon, G-CSF = granulocyte-colony stimulating factor, LIF = leukemia inhibitory factor, ENA = epithelial-derived neutrophil-activating peptide, ICAM = intercellular Adhesion Molecule, MIP = macrophage inflammatory protein, MCP = monocyte chemoattractant protein, VEGF = Vascular endothelial growth factor, NGF = nerve growth factor, SCF = stem cell factor, CD = cluster of differentiation, VCAM = vascular cell adhesion molecule, HGF = hepatocyte growth factor, TRAIL = TNF-related apoptosis-inducing ligand, GM-CSF = granulocyte macrophage colony-stimulating factor, PIGF = placental growth factor, IP = interferon γ-induced protein, PAI = plasminogen activator inhibitor, EGF = epidermal growth factor, RANTES = regulated on activation, normal T cell expressed and secreted, SDF = stromal cell-derived facto, BDNF = brain-derived neurotrophic factor, PDGF = platelet-derived growth factor, MIG = monokine induced by gamma interferon, GRO-α = growth regulated α protein, MCSF = macrophage colony stimulating factor, FGF = fibroblast growth factor , FasL = fas ligand, s.e. = standard error.

Outcome Variable	>50% Detectable	Lowest Detectable pg/mL	BL Phase Mean ± s.e. pg/mL	Drug Phase Mean ± s.e. pg/mL	Change BL to Drug	
					*F*	*p*
Cytokines						
IL-6	3	8.8	15.0 ± 5.7	9.7 ± 5.7	31.974	<0.001
IL-12p70	8	6.34	2.5 ± 0.5	2.2 ± 0.5	15.540	<0.001
TNF-α	8	9.38	146.9 ± 30.6	138.4 ± 30.6	11.381	0.002
IL-15	8	2.57	25.5 ± 12.5	21.9 ± 12.5	10.418	0.002
IL-17A	8	2.35	8.4 ± 2.9	7.1 ± 2.9	10.139	0.003
IL-12p40	8	1.27	6.9 ± 0.7	6.5 ± 0.7	9.124	0.004
TGF-β	6	1.28	38.2 ± 22.5	32.7 ± 22.5	8.499	0.006
IL-1Ra	1	31.53	2446.5 ± 64.6	1995.2 ± 50.5	17.931	0.008
IL-10	4	1.76	22.7 ± 10.8	16.5 ± 10.8	8.472	0.008
IL-27	2	22.67	60.3 ± 34.8	36.1 ± 35.2	8.405	0.016
IL-5	2	9.99	6.1 ± 3.9	1.0 ± 4.0	8.351	0.016
IFN-α	8	0.5	6.2 ± 1.4	5.5 ± 1.4	8.089	0.007
IL-4	8	7.52	29.5 ± 8.9	25.5 ± 8.9	8.088	0.007
IL-1β	7	2.07	2.3 ± 1.5	1.5 ± 1.5	7.414	0.010
IL-2	4	4.33	30.0 ± 15.1	22.7 ± 15.2	6.921	0.015
G-CSF	8	7.17	50.2 ± 14.6	45.9 ± 14.5	6.918	0.012
TGF-α	5	0.97	1.1 ± 0.3	0.7 ± 0.3	6.663	0.016
LIF	8	4.69	16.7 ± 9.8	15.2 ± 9.8	5.389	0.025
ENA78 (CXCL5)	8	5.31	92.7 ± 26.2	72.9 ± 26.0	5.101	0.030
IFN-γ	8	3.69	12.7 ± 2.5	11.3 ± 2.5	4.928	0.032
IFN-β	8	10.44	68.1 ± 23.6	58.3 ± 23.5	4.590	0.038
ICAM-1 (CD54)	8	5.24	2682.9 ± 354.9	2544.5 ± 353.8	4.564	0.038
IL-13	4	1.93	1.9 ± 1.2	1.3 ± 1.2	4.469	0.047
MIP-1α (CCL3)	6	1.34	19.1 ± 9.4	16.8 ± 9.4	4.464	0.042
Resistin	8	5.97	1970.8 ± 178.5	2113.7 ± 176.1	4.410	0.041
MCP3 (CCL7)	8	9.3	35.1 ± 7.3	30.7 ± 7.2	3.592	0.065
VEGF	8	5.89	175.8 ± 79.0	159.8 ± 78.9	3.514	0.067
NGF	5	4.89	35.3 ± 25.7	29.6 ± 25.7	3.438	0.080
IL-18	8	10.53	7.6 ± 1.6	6.7 ± 1.6	3.053	0.088
SCF	4	1.54	20.1 ± 14.1	18.4 ± 14.1	2.638	0.119
MCP-1 (CCL2)	8	0.67	38.4 ± 9.1	33.9 ± 9.0	2.409	0.128
IL-17F	8	1.89	3.3 ± 1.3	3.0 ± 1.3	1.760	0.191
CD40L (CD154)	8	5.45	94.5 ± 37.5	85.8 ± 37.4	1.685	0.201
IL-7	8	0.47	11.0 ± 0.9	11.0 ± 0.9	1.601	0.212
MIP-1β (CCL4)	4	4.11	95.4 ± 60.1	90.2 ± 60.1	1.417	0.248
VCAM1 (CD106)	8	12.16	34,309.8 ± 4074.2	36,409.7 ± 3989.6	1.192	0.281
HGF	8	10.19	409.5 ± 175.1	384.2 ± 174.8	0.985	0.326
Eotaxin (CCL11)	8	0.7	38.5 ± 7.6	37.0 ± 7.5	0.893	0.350
TRAIL	8	6.51	133.7 ± 114.0	139.2 ± 114.0	0.718	0.402
GM-CSF	8	31.86	10,818.8 ± 3481.4	10,220.5 ± 3466.6	0.645	0.426
IL-21	3	10.19	230.5 ± 209.5	220.6 ± 209.6	0.537	0.478
PIGF-1	1	1.04	2.0 ± 0.1	2.1 ± 0.1	0.506	0.508
IL-1α	1	0.57	3.8 ± 42.1	4.1 ± 42.1	0.358	0.576
IP-10 (CXCL10)	8	1.99	5.9 ± 0.6	5.7 ± 0.5	0.348	0.558
PAI1	8	24.29	17,337.7 ± 1721.5	17,574.4 ± 1710.0	0.263	0.610
EGF	6	2.45	10.7 ± 5.3	7.9 ± 4.7	0.225	0.639
RANTES (CCL5)	8	0.83	63.9 ± 8.6	64.6 ± 8.6	0.202	0.655
SDF1a (CXCL12)	8	6.32	365.2 ± 230.8	359.9 ± 230.7	0.142	0.708
VEGF-D	7	11.14	13.0 ± 7.5	12.6 ± 7.5	0.100	0.753
Leptin	8	23	4707.2 ± 1114.9	4676.1 ± 1110.6	0.018	0.893
BDNF	8	1.73	821.0 ± 155.9	827.2 ± 151.2	0.005	0.945
PDGF-BB	8	7.62	175.1 ± 24.7	176.0 ± 23.9	0.004	0.948
MIG (CXCL9)	8	9.08	230.4 ± 183.0	230.1 ± 182.9	0.002	0.962
IL-22	6	29.43	308.1 ± 112.1	310.6 ± 109.8	0.002	0.967
IL-31	0	18.95	-	-	-	-
IL-9	0	9.3	-	-	-	-
TNF-β	0	1.15	-	-	-	-
GRO-α (CXCL1)	0	1.66	-	-	-	-
IL-23	0	20.03	-	-	-	-
MCSF	0	21.1	-	-	-	-
FGF-β	0	4.68	-	-	-	-
FasL	0	2.14	-	-	-	-
IL-8 (CXCL8)	0	1.53	-	-	-	-
Symptoms						
Pain			51.1 ± 4.8	43.3 ± 4.8	13.998	<0.001
Overall symptoms			55.7 ± 5	45.5 ± 4.6	23.341	<0.001
